# Bidirectionality of smoking and depression in adolescents: a systematic review

**DOI:** 10.47626/2237-6089-2021-0429

**Published:** 2023-06-27

**Authors:** Mudassir Farooqui, Samra Shoaib, Humera Afaq, Syed Quadri, Fatima Zaina, Aqsa Baig, Ayesha Liaquat, Zoona Sarwar, Atif Zafar, Sana Younus

**Affiliations:** 1 Department of Neurology University of Iowa Hospitals and Clinics Iowa City IA USA Department of Neurology, University of Iowa Hospitals and Clinics, Iowa City, IA, USA.; 2 Department of Psychiatry Nassau University Medical Center East Meadow NY USA Department of Psychiatry, Nassau University Medical Center, East Meadow, NY, USA.; 3 Department of Public Health National University San Diego CA USA Department of Public Health, National University, San Diego, CA, USA.; 4 Department of Neurology Massachusetts General Hospital Harvard Medical School Boston MA USA Department of Neurology, Massachusetts General Hospital, Harvard Medical School, Boston, MA, USA.; 5 Department of Pulmonology Ziauddin University and Hospital Karachi Pakistan Department of Pulmonology, Ziauddin University and Hospital, Karachi, Pakistan.; 6 Liaquat National Hospital Medical College Karachi Pakistan Liaquat National Hospital & Medical College, Karachi, Pakistan.; 7 Karachi Medical and Dental College Karachi Pakistan Karachi Medical and Dental College, Karachi, Pakistan.; 8 Department of Surgery University of Oklahoma Oklahoma City OK USA Department of Surgery, University of Oklahoma, Oklahoma City, OK, USA.; 9 Department of Neurology University of Toronto Toronto ON Canada Department of Neurology, University of Toronto, Toronto, ON, Canada.; 10 Menninger Department of Psychiatry and Behavioral Sciences Baylor College of Medicine Houston TX USA Menninger Department of Psychiatry and Behavioral Sciences, Baylor College of Medicine, Houston, TX, USA.

**Keywords:** Smoking, depression, adolescents, mental health, depressive disorder

## Abstract

**Introduction:**

Recently, evidence has been accumulating that both smoking and mental health disorders are continuously increasing among adolescents. This systematic review elucidates the research into evidence of the direction of the association and risk factors influencing the relationship between smoking and depression. We also highlight recent studies on the effects of electronic cigarettes and developments on the association between depression and smoking.

**Methods:**

A literature search was conducted on databases including PubMed, Ovid Medline, EMBASE, and PsycINFO and in relevant neurology and psychiatry journals. Terms used for electronic searches included smoking, tobacco, cigarettes; depression; adolescent, youth; direction. Relevant information was then utilized to synthesize findings on the association between smoking and depression among adolescent population.

**Results:**

The initial database searches yielded 2,738 related articles. After screening and cross-referencing, duplicate articles, articles published in languages other than English, and studies on animals, social and lifestyle factors, mood disorders, and substance use were excluded. Of these, a total of 122 publications only focusing on smoking and depression in the adolescent population were selected for synthesis in this qualitative systemic review. These include 110 original research articles, eight meta-analyses and reviews, and four reports and websites.

**Conclusion:**

The relationship between smoking and depression in the literature does not reflect the cause-effect relationship. The lack of evidence on the direction of the association may reflect futile study designs, confounding factors and/or use of indirect measures of depression and quantification of smoking. Future prospective randomized studies should target elucidation of the causal association.

## Introduction

Tobacco use is one of the leading causes of preventable deaths in the United States. According to the Centers for Disease Control and Prevention (CDC), 480,000 Americans die of cigarette smoking each year and smoking related illnesses cost about 300 billion dollars every year.^1^ The U.S. Department of Health and Human Services report *Health consequences of smoking* states that tobacco use and addiction mostly begins in early youth or adulthood.^2^ About 87% of smokers begin smoking in their teens and become regular smokers in adulthood.^2^ According to the 2012 surgeon general’s report *The health consequences of smoking-50 years of progress* , there were approximately 1.2 million adolescent smokers in the United States.^2^ While in 2015, the CDC reported an estimated 4.7 million middle and high school adolescent students were current tobacco product users.^3^ Each day more than 3,000 teenagers start smoking and around 2,000 adolescents and young adults who are occasional smokers become daily smokers.^2 , 3^ It is estimated that with the current national smoking trends around 5.6 million current adolescent smokers will die prematurely due to smoking.^2^

Mental health of adolescents has been a growing concern in the U.S. population. According to the National Institute of Mental Health (NIMH), in 2017, an estimated 3.2 million adolescents aged 12-17 in the United States had had at least one major depressive episode in the past year.^4^ This represents 13.3% of the U.S. population aged 12-17. Adolescent females have a higher prevalence (20%) of depression than adolescent males (6.8%).^4^ In the United States, from 2009-2017, rates of depression increased by more than 60% while suicide rates due to depression including suicide ideation, attempt, and/or deaths doubled among young adolescents.^5^ An association between smoking and depression has been examined by researchers with emphasis on initiation and progression of smoking and its relationship with depression. The aim of this review is to explore these complex dynamics and discuss the associated factors influencing the relationship with depression among smokers in the adolescent population.

## Methods

An extensive review was conducted of the literature published since 1990 using biomedical databases (PubMed, Ovid Medline, EMBASE, and PsycINFO) to seek peer-reviewed articles using the following keywords: smoking, tobacco, cigarettes; depression; adolescent, youth; direction. Cross-checking of references enabled identification of additional relevant articles. Articles related to the keywords were thoroughly searched to exclude studies on animals, social and lifestyle factors, mood disorders, and substance use, selecting only articles focusing on smoking and depression in the adolescent population. Duplicate articles in these databases and full-text articles in languages other than English were also screened and excluded. The authors took all decisions on whether to include or eliminate relevant articles and performed all data extraction and any controversies or disagreements were settled by discussion. The qualitative results are reported according to the Preferred Reporting Items for Systematic Reviews and Meta-Analyses (PRISMA) guidelines.

## Results

**Figure 1:**
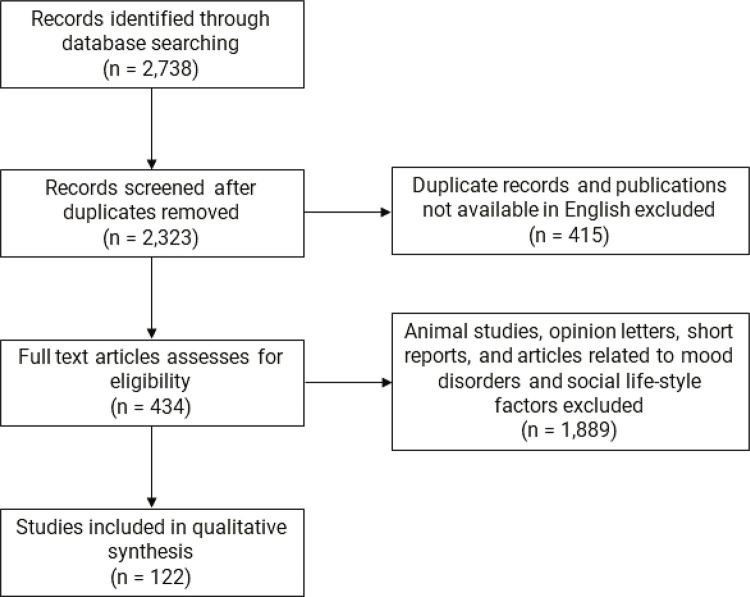
Article eligibility flowchart. Exclusion criteria: 1) duplicate articles; 2) articles not in English; 3) opinion letter or short review; 4) animal studies; and 5) articles on mood disorders, and social and lifestyle factors.

The initial search of the databases yielded 2,738 related articles. After cross-referencing, duplicates were excluded and articles were screened for availability of full text in English. Studies on animals, social and lifestyle factors, mood disorders, and substance use were then excluded from among the 2,323 full-text articles available. Screening of the titles and abstracts yielded 434 pertinent articles. Irrelevant articles and narrative reviews were then excluded, leaving a total of 122 pertinent publications that were used in the synthesis of this qualitative systematic review. These articles comprise 110 original research articles, eight meta-analyses and reviews, and four reports and websites. The relevant available information from these articles was then used to describe the clinical characteristics of depression, risk factors, and the relationship between smoking and depression in the adolescent population ( Figure 1 ).

### Clinical characteristics of depression

The American Psychiatric Association’s (APA) Diagnostic and Statistical Manual of Mental Disorders, 5th edition (DSM-5) describes depression as a serious mental health disorder. Individuals with depression may feel sad, tired, or worthless and lack motivation to do anything. Some may also have difficulty thinking clearly and may have suicidal thoughts. Diagnostic criteria are met if the symptoms have been present for at least 2 weeks, but the severity of the disorder is based upon the number of symptoms present at the time of presentation^6^ ( Table 1 ).

**Table 1:** Symptoms of depression

Typical symptoms	Related symptoms
Unresponsive depressed mood	Decreased concentration
Loss of interest and enjoyment	Loss of self-esteem and self-confidence
Decreased energy/activity (increased fatigability and tiredness)	Feelings of guilt and unworthiness
	Negative views of future
	Self-harm or suicidal behavior
	Deranged sleep pattern
	Psychomotor agitation or retardation
	Loss of appetite and weight loss (≥ 5% body weight in past month)
	Loss of libido

Mild episode = presence of two typical symptoms and at least two related symptoms.

Moderate episode = presence of at least two typical symptoms and three or four related symptoms.

Severe episode = presence of all three of the typical symptoms along with at least four related symptoms, some of which should be of severe intensity.

### Risk factors for depression

#### Genetics

Studies have demonstrated genetic variance of 40-80% as a risk factor for the development of depressive disorder.^7 , 8^ Twins have an overall significantly higher rate of depression than the general population with greater concordance among monozygotic than dizygotic twins.^9^ The heritability of brain size, various characteristics of brain shape, and regional brain volume as related to the development of depression is still under scrutiny, but functional and structural imaging studies have identified specific brain circuits which are involved in the development of this disorder.^10^

#### Cognitive

Depressive symptoms at an early age are a risk factor for development of depression and incapacitated social behavior at a later period in life.^11^ Stressful life, adverse events, and negativity in emotions and lifestyle contribute significantly to development of depression in adolescence.^12^ These stressful events in life are linked to serotonin transporter gene polymorphism, resulting in initiation and exacerbation of depressive symptoms; thus demonstrating an interaction between genetic sequences and the development of depression.^13^

#### Social factors

Parental depression, family disintegration, and parent-children conflicts strongly contribute to development of depressive psychopathology.^14^ Social factors like academic performance and relationships with peers can be important triggers of the development of depression at an early stage in life.^15 - 18^ Traumatic upbringing, sexual, emotional, or physical abuse and the experience of losing loved ones also play an important part in the development of depression later in life. Individuals with a history of sexual abuse, specially involving intercourse, pose one of the greatest risks for the development of depression.^18 - 20^ Adolescent victims are at an increased risk of developing a depressive disorder.^21 , 22^ Emotional abuse and/or excessive criticism from parents is also associated with depressive symptoms in adolescents.^23^

## Risk factors for smoking

### Sociodemographic factors

Factors like age, socioeconomic status, sex, and ethnicity were found to be associated with smoking initiation and continuation. Sex is a significant contributor, although some studies found that females were prone to smoking initiation, whereas others report male sex to have an increased risk.^24 , 25^ Similarly, ethnicity has also been significantly associated with smoking behavior; some researchers suggest whites are more at risk while others identify Hispanics and blacks.^26 , 27^ These relationships between race and ethnicity and smoking behavior are intertwined with and influenced by social and environmental factors like poverty, family environment and smoking status, peer attitudes, school and lifestyle exposure, and other psychological factors.^28 - 30^

### Psychological factors

Researchers have implicated personality traits like impulsivity, sensation seeking, risk taking, and/or rebelliousness as being associated with smoking onset.^31 - 33^ Others report that negative characteristics including academic performance, self-esteem, depression or distress, and coping behavior (including anger and anxiety) impact the relationship of smoking initiation in young adolescents.^34 - 36^

### Personal and environmental factors

The social environment, including smoking by parents, siblings, family members, friends, and peers, has been strongly implicated in smoking initiation among young adolescents.^37 - 39^ Some studies concluded that parental smoking has an increased effect on girls compared to boys, while others report that poor relationship among parents significantly affects the prevalence of smoking initiation.^40 , 41^ Dose dependent relationships and attitude to smoking behavior of parents, siblings, and friends have also been shown to influence smoking initiation among young adolescents.^42 , 43^ On the other hand, parental monitoring/supervision was observed to protect children from smoking behavior.^44^ Other social factors like physical activity, receptivity to tobacco promotions, marketing, and advertising were also reported to influence smoking initiation.^44 , 45^

Researchers have postulated various mechanisms to elucidate the complex relationship between smoking and depression. Some argue in support of the “self-medication” theory, suggesting that depressed individuals initiate smoking to control or alleviate their mood symptoms.^46 , 47^ It is postulated that nicotine acts as an antidepressant in these cases by releasing neurotransmitters like dopamine, norepinephrine, and acetylcholine.^46^ Other researchers suggested that smoking increases stress levels which in turn contribute to development of depression over time.^48^ Many researchers propose that the relationship is bidirectional; stress or adverse events trigger initiation of smoking, resulting in development of depression, which leads to neurochemical changes in the brain, influencing smoking behavior.^49^ The association is further complicated by risk factors like genetics and social stressors which may also influence the development of both depression and smoking simultaneously.^50 , 51^

## Smoking causing depression

Many studies support smoking as a potential precursor for the development of depression. In their prospective cohort studies, Wu et al.^52^ and Goodman et al.^53^ observed that cigarette smoking leads to depressive symptoms over time (hazard ratio [HR] 1.66 95% confidence interval [95%CI] 1.28-2.16 and odds ratio [OR] 3.90 95%CI 1.85-8.20, respectively, when compared to non-smokers). Similarly, Martini et al.^54^ reported a gradient association between smoking and the severity of depressive symptoms; where depressive symptoms were more pronounced in current smokers, followed by former smokers, compared to non-smokers, while female smokers were observed to have more depressive symptoms than male smokers. Steuber et al.^55^ investigated the direction of the relationship between smoking and depression in adolescents and also reported an increase in depressive symptoms in females during the period of smoking initiation. They reported increases in the likelihood of depressive symptoms in three groups of adolescents including starters (1.5 times), quitters (1.4 times), and maintainers (2.0 times), compared to their contemporaries who had never smoked. Moreover, Boden et al.^51^ observed that an increase in the level of nicotine dependence (based on nicotine dependence symptoms) was positively associated with increased depressive symptoms (p < 0.005). Adolescents who reported at least five symptoms of nicotine dependence were found to have the highest rates of depressive symptoms (2.13 times, 95%CI 1.98-2.31) when compared with non-nicotine dependent adolescents with no symptoms. Although these studies were able to demonstrate an association between smoking and depressive symptoms, some of them were unable to establish the reverse association of depression being the antecedent to smoking behavior among adolescent populations^52 , 54 - 68^ ( Table 2 ).

**Table 2:** Studies showing the association of smoking causing depression in adolescents

First author and year	Country	Study design	Sample size	Main findings
Wu ^52^ 1999	United States	Population based prospective cohort study	1,731	This study was conducted by the Johns Hopkins University on public school students from the mid-Atlantic area aged 8-9 to 13-14 and consisting of an almost equal percentage of male and female subjects, with 75% being African Americans. It was concluded that smoking behaviors were associated with an increased risk for the onset of depression, but the presence of depressive symptoms did not lead to an increased potential for smoking initiation.
Steuber ^55^ 2006	United States	National Longitudinal Study of Adolescent Health	14,634	Adolescent smokers, quitters, and maintainers were more likely to be depressed as compared to their peers who had never smoked. Females were observed to have increased depressive symptoms around the time of onset of smoking.
Raffetti ^56^ 2019	Sweden	Longitudinal study	3,959	Current cigarette smoking, snus use, and tobacco dependence were assessed using questionnaires at baseline and 1-year follow-up. The outcome was onset of depressive symptoms measured with the CES-DC scale. The incidence of depressive symptoms at follow-up was greater in current than never smokers at baseline. Current cigarette smoking at the age of 13 years was strongly associated with onset of depressive symptoms 1 year later, with a significant interaction between tobacco use and sex. Feeling dependent on tobacco was also associated with depressive symptoms in males but not in females. Snus and overall tobacco use were not associated with the onset of depressive symptoms.
McKelvey ^60^ 2017	United States	Longitudinal survey	176	Adolescent cigarette smokers completed surveys assessing drug use, smoking characteristics, demographics, and depressive symptoms at baseline and at 12, 24, and 36 months follow-up. Participants reported using, on average, two substances in addition to cigarettes. Adolescent cigarette-smokers who reported extended range use also reported symptoms of clinical depression at baseline and follow-up.
Ranjit ^69^ 2019	United States	Longitudinal study of adolescent twins	4,152 individuals, 1,910 twin pairs	Longitudinal study evaluating the association between cigarette smoking and development of depressive symptoms among adolescents in a Finnish twin cohort. It was observed that cigarette smoking at the age of 14 predicted depressive symptoms at the age of 17. Regular smokers had higher depression scores as compared to never smokers. Within pairs, the estimates were lower for monozygotic pairs compared to dizygotic pairs, suggesting that shared genetic factors contribute to the associations observed in individual-based analyses.
Lee ^70^ 2019	South Korea	Longitudinal study	62,276	This study was conducted with a Korean adolescent population to assess the association of depression and suicidality with conventional and e-cigarette use. Dual users had a higher prevalence of depression and suicidality for both lifetime and current use; e-cigarette-only users had higher levels of depression and suicidality than nonusers; and among female adolescents, conventional-cigarette-only users, e-cigarette-only users, and dual users had a higher prevalence of depression and suicidality than male adolescents
Goodman ^53^ 2000	United States	Longitudinal survey	First sample 8,704; second sample 6,947	Baseline and 1-year follow-up data from the National Longitudinal Study of Adolescent Health. Current cigarette smoking was the strongest predictor of developing high depressive symptoms, whereas baseline high depressive symptoms did not predict smoking behavior.
Beal ^71^ 2014	United States	Longitudinal	262	This study examined the associations between smoking and depressive symptoms across ages 11 to 20. Increased smoking predicts a greater increase in depressive symptoms across adolescence.
Boden ^51^ 2010	New Zealand	Longitudinal	1,055	A birth cohort was followed, and data were collected at birth, 4 months, 1 year, and annually to age 16 years, and again at ages 18, 21, and 25 years. Nicotine dependence and depression were assessed in adolescence and were observed to be associated at ages 18, 21, and 25 years. Among other models, nicotine dependence leading to increased risk of depression was found to be the best fitting causal model.
Schuler ^72^ 2015	United States	Longitudinal survey	6,070	Association between substance use behaviors and depressive symptoms were measured during young adulthood using time-varying effect models. Marijuana use and daily smoking were significantly associated with depressive symptoms at most ages from 12 to 31. There were no gender differences observed during adolescence.

CES-DC = Center for Epidemiological Studies Depression Scale for Children.

Studies have also explored this relationship while observing the effects of substance abuse. Schuler et al.^72^ estimated age-related trends to observe the association between behavior and depression using time varying models. They concluded that daily smoking and substance use is strongly related to depressive symptoms, during both the adolescent and adult phases, from ages 12 to 31 years, whereas drinking behavior was only associated during adolescence. However, another longitudinal study by Gage et al.^73^ was not able to ascertain the presence of depression and smoking at the age of 18 years among adolescents smokers recruited at the age of 16 years, while controlling for substance abuse.

Researchers have also found that second-hand smoke (SHS) is also associated with development of depressive symptoms among adolescents. In their cross sectional study, Lee et al.^74^ reported that SHS exposure is associated with depression (OR: 1.27) in both males (OR: 1.52) and females (OR: 1.72). Bandiera et al.^75^ used National Health and Nutrition Examination Survey (NHANES) data to observe that SHS is significantly associated with major depressive disorder (b = 0.22) among adolescents. Similarly, in their study of data from the Korea Youth Risk Behavior Web-based Survey, Bang et al.^76^ observed that SHS significantly increased the odds of depression (OR: 1.339) among adolescents aged 12-18 years. A recent global study by Jacob et al.^77^ using Global School-Based Student Health Survey data from 22 countries reported a dose-dependent increase in depressive symptoms (1-2 days: OR: 1.06; 3-6 days: OR:1.38; 7 days: OR: 1.63) among school-going adolescents aged 13-15 years.

## Depression causing smoking

Individuals with mental health disorders usually start smoking at a younger age and are more dependent on nicotine than the general population.^78^ McKenzie et al.^79^ estimated a two-fold increase in the odds of nicotine dependence in adolescent individuals with high levels of depression and anxiety. Similarly, Lenz et al.^80^ observed that adolescents with some existing depressive symptoms are more prone to use tobacco. Khaled et al.^64^ conducted a study to determine the impact of depression on smoking. They observed that depressed adolescents have an increased risk of smoking under stress (HR of time to first cigarette smoked after waking: 1.7, 95%CI 1.1-2.5). Although, some studies have reported depression and its association with smoking,^81 - 86^ Redner et al.^87^ were not able to ascertain the association between depression and smokeless tobacco use (OR: 0.90, 95%CI 0.54-1.49) among adolescent smokeless tobacco users.

A longitudinal study by Bares et al.^88^ observed a positive association between depressive symptoms and subsequent increase in cigarette use in adolescents. However, the authors were not able to establish the reverse relationship among the male population. Moreover, a study by Morrell et al.^89^ investigating college students found that young females with depressive vulnerability smoked to relieve their mood symptoms. Although some of these longitudinal and cross-sectional studies were able to establish strong associations with depressive symptoms leading to smoking behavior, other studies were not able to observe findings to support this hypothesis.^52 , 53^

Researchers have also studied the influence of societal and environmental factors on smoking initiation and depression among adolescents. In a prospective cohort of adolescents aged 14-15, Patton et al.,^68^ evaluated depression and anxiety and reported peer smoking as a strong predictor for smoking initiation (HR 6.7, 95%CI 3.4-13). Bullying and hostility among young individuals is also seen to increase risk of developing depression and smoking behavior.^90 - 92^ Whalen et al.^93^ also observed that aggression, anger, and/or sadness are often linked with initiation of smoking in adolescence. Smoking initiation was observed to be a coping mechanism for stress, frustration, depression, and anxiety.^93 , 94^ Moreover, Tercyak et al.^95^ reported that depressed adolescents were more responsive to tobacco advertising (OR 1.25, 95%CI 1.02-1.53) and therefore were more likely to start smoking. Adolescent smokers also tend to show behavior disorders like conduct disorder and attention deficit conduct disorder (ADHD) along with depression and are prone to use other substances of abuse later in life^80 , 96 , 97^ ( Table 3 ).

**Table 3:** Studies showing the association of depression causing smoking in adolescents

First author and year	Country	Study design	Sample size	Main findings
Khaled ^64^ 2011	Canada	NPHS	13,298	Canadian adolescents > 12 years old were included to measure the determinants of associated factors between smoking and depression. Individuals with depressive symptoms were found to have shorter time to first cigarette, within 5 min of waking up, and were more inclined to smoke during times of stress.
Lam ^65^ 2005	Hong Kong	Longitudinal	1,894	Associations between depressive symptoms and smoking were evaluated among adolescents at baseline and 12 months later. Both current and former smokers had higher depressive symptoms than never smokers. Smoking at baseline predicted depressive symptoms. Among non-smokers, depressive symptoms at baseline predicted smoking at 12 months. Persistent, past, and new smokers had higher depressive symptoms at both waves than non-smokers. Depressive symptoms promote tobacco use in Asian adolescents by making it more likely that an adolescent will begin smoking and less likely that she or he will quit.
Windle ^67^ 2001	United States	Four-wave longitudinal study	1,218	The study measured directionality of the association between cigarette smoking behavior and depressive symptoms over time. Increased and persistent depressive symptoms predicted increased cigarette use. Similarly, heavy and persistent smoking prospectively predicted increases in depressive symptoms.
Patton ^68^ 1998	Australia	Six-wave longitudinal cohort	2,032	This cohort study analyzed 14 and 15-year-old secondary school students in Australia, concluding that adolescents who smoked experimentally were 29 times more likely than non-smokers to adopt smoking as a habit in the succeeding half year. This study also determined that anxiety and depression led to higher likelihood of initiation of cigarette smoking with peer pressure adding modestly to the risk.
Windle ^82^ 2018	United States	Longitudinal cohort – Web-based surveys	2,969	The survey’s findings indicate that more adverse childhood experiences prior to 18 years of age are associated with higher levels of depressive symptoms, ADHD symptoms, cigarette use, alcohol use, marijuana use, and BMI, in addition to lower levels of fruit and vegetable intake, and sleep.
Fergusson ^84^ 2003	New Zealand	Longitudinal study	1,265	Data were collected on the birth cohort for the follow up period of 16-21 years. It was concluded that major depression was associated with increased rates of daily smoking and nicotine dependence.
McKenzie ^79^ 2010	Australia	Longitudinal	1,943	Data were collected in two follow-up assessments during young adulthood at 6-monthly intervals. Adolescents with high levels of depression and anxiety symptoms had two-fold higher odds of nicotine dependence in young adulthood compared to young adults with low levels of adolescent depression and anxiety symptoms.
Bandiera ^98^ 2017	United States	Longitudinal survey	5,445	This study was conducted across 1 year to evaluate the bidirectional relationship between electronic cigarette use and elevated depressive symptoms among college students. Depressive symptoms predicted electronic cigarette use at 6 months and 1 year, whereas electronic cigarette use did not predict elevated depressive symptoms at 6 months or 1 year.

ADHD = attention deficit conduct disorder; BMI = body mass index; NPHS = National Population Health Survey.

## Bidirectionality

Although several of the studies above have discussed the relationship of smoking and depression as antecedents of one another, this complex association is multifaceted and many researchers have tried to explore the idea of bidirectionality. Windle et al.^67^ studied 1,218 school teenagers, concluding that regular heavy smoking significantly increases depressive episodes while persistently elevated depressive symptoms results in increased smoking behavior. Similarly, a study that Orlando et al.^99^ conducted with school-going adolescents observed that emotional distress in grade 10 was associated with an increase in smoking behavior in grade 12 and smoking in grade 12 subsequently resulted in an increase in the emotional distress during young adulthood. They observed a similar pattern among boys and girls; but smoking initiation during young adulthood was increased among boys compared to girls. Conversely, in a longitudinal study of healthy girls aged between 11 and 17, Beal et al.^71^ concluded that an increased prevalence of cigarette smoking was associated with an increase in depressive symptoms in young girls across time. However, they were not able to conclude the reverse association of depressive symptoms leading to an increase in smoking.

The bidirectional relationship was also explored by McGovern et al.,^47^ who followed an adolescent cohort of 1,093 students for 4 years to assess the relationship between smoking and depression across time. They reported that depressive symptoms in early adolescence are a precursor to smoking behavior during late adolescence, whereas an increase in smoking predicts suppression of these depressive symptoms. These effects were mediated by peer smoking. In contrast, Brook et al.^100^ followed 688 teenagers for 13 years and reported that early cigarette smoking during adolescence is a predictor of depressive symptoms in adulthood, but the reciprocal association with history of depressive symptoms was not statistically significant as a precursor for smoking behavior ( Table 4 ).

**Table 4:** Selected studies of the bidirectional association of smoking with depression in adolescents

First author and year	Country	Study design	Sample size	Main findings
Weiss ^66^ 2005	United States	Longitudinal survey	1,699	Data were collected from adolescents in the study at the start of grade 6 and in the 7th grade. Among 6th graders who had never smoked, depressive symptoms and hostility played a part in increasing the risk of smoking behavior by the time they reached 7th grade. Students with prior experience of smoking, increases in depressive symptoms, and hostility were associated with more frequent smoking.
Bares ^88^ 2014	United States	Bivariate auto-regressive multi-group structural equation model	6,501	This study demonstrated bidirectionality between depressive symptoms and cigarette use among adolescent females only. The association was not observed for adolescent male participants.
Escobedo ^85^ 1999	United States	Longitudinal survey	7,885	This nationally representative sample survey was conducted to measure the association of smoking with depression. Depressed adolescents were more likely to start smoking as compared to non-depressed individuals. Increased frequency of depressive symptoms increased the rates of smoking initiation.
Wilkinson ^81^ 2016	United States	National Longitudinal Study of Adolescent to Adult Health	12,017	Data from the National Longitudinal Study of Adolescent to Adult Health were used to investigate longitudinal associations between high frequency substance use (alcohol, cigarettes, and marijuana) and depressive symptoms. Increases in depressive symptoms were associated with a later increase in frequency of marijuana use for males and an increase in smoking frequency for females. Equally, increases in smoking frequency were significantly associated with approximately a 0.6-point increase for females and a 0.4-point increase for males in depressive symptoms at a later wave. Results indicate a bidirectional relationship between smoking and depressive symptoms for females
Leung ^63^ 2011	Australia	Longitudinal survey	10,012	Longitudinal study of adolescent women with follow-up over 13 years showed that incidence of depression was higher among smokers and that depressed individuals were more likely to initiate smoking.
Lechner ^101^ 2017	United States	Longitudinal survey	2,460	This study was conducted to demonstrate associations between e-cigarette and combustible cigarette use and mental health symptomatology. Adolescents who had never previously used combustible or e-cigarettes were assessed at baseline and at 6 and 12-month follow-ups. Higher baseline depressive symptoms predicted subsequent onset of cigarette and e-cigarette use and dual use of both products. Sustained use of e-cigarettes over the 12-month observation was associated with a greater rate of increase in depressive symptoms over time. Among those who sustained use of e-cigarettes, higher frequency of use was associated with higher depressive symptoms at the final follow-up.
Brown ^86^ 1996	United States	Longitudinal survey	1,709	Adolescents were surveyed on two occasions, 1 year apart. Smoking increased the risk of developing major depressive disorder and drug abuse/dependence. Prevalence of major depressive disorder predicts onset of smoking.
Brook ^100^ 2004	United States	Longitudinal survey	688	Earlier cigarette smoking in adolescence predicts later depressive symptoms in the late twenties. Depressive symptoms during adolescence predict cigarette smoking in the late twenties but not above and beyond prior smoking.

## Bidirectionality and social factors

The relation between smoking and depression is very complex. Much research has been done to determine the direction of association. However, this intricate connection remains obscure due to its multifactorial effects. Researchers have investigated the social factors that influence this relationship, especially in adolescents. Horton et al.^102^ observed an increased effect of smoking on depressive symptoms in adolescent females with either negative or high level of positive religious coping behavior, whereas moderate religious coping decreased the likelihood of smoking. Romantic breakups, interpersonal stress and family disruption, and decreased church attendance were also found to be associated with mental health issues including depressive episodes, along with substance abuse and smoking among adolescents.^103 , 104^

Peer pressure significantly mediates the association between smoking and depression, especially in adolescent females compared to males.^105^ Hostility and rebelliousness were also found to be associated with smoking behavior and the latter was also reported to be a significant predictor of development of depression in adolescents.^66 , 106^ Parental attitude including smoking by either parent or living with a single parent or guardian, low self-esteem, difficulty coping with stress, low grades in school, social isolation and withdrawal, sense of helplessness, and friends with smoking behavior have also been instrumental in the development of the relationship between smoking and depression in adolescents.^107 , 108^

Smoking in adolescence is also associated with both internalizing and externalizing problems; the observation that young smokers may use smoking behavior to improve the mood symptoms and self-medicate themselves with nicotine to rescue higher functioning has been proposed as a plausible interpretation of the behavior rationale among the adolescent population^109 , 110^ ( Table 5 ).

**Table 5:** Selected studies of the association of social and demographic factors with smoking and depression in adolescents

First author and year	Country	Study design	Sample size	Main findings
McGovern ^111^ 2004	United States	Longitudinal study assessing the genetic effects of smoking	615	The effects of smoking on dopamine transporter (SLC6A3) and dopamine receptor (DRD2) were evaluated, observing that presence of the DRD2 A1 allele significantly increases the probability of smoking in adolescent smokers.
Gibbons ^57^ 2018	United States	Longitudinal study	889	A prospective study was conducted to observe the relationship between PRD and smoking in African American children. PRD was assessed at ages ranging from 10.5 to 24.5. Anger and depressive symptoms were assessed at age 12.5 and at age 24.5. Early PRD predicted smoking at later ages. Negative effects mediated this association while cultural socialization was associated with lower rates of adolescent smoking and buffered the relation between PRD and anger.
Roohafza ^61^ 2017	Isfahan	Self-administered questionnaire	5500	Data were collected with the help of a self-administered questionnaire on background characteristics, smoking status, depression, and risk factors. Lower education attainment of fathers was accompanied by higher depression prevalence in adolescents. Parental smoking and sibling smoking increased the depression likelihood for never-smokers. Positive attitude toward smoking increased the probability of depression among never-smokers. A higher level of self-efficacy was related to lower chance of depression. Risky behavior increased depression likelihood in never-smokers, in experimental smokers, and in current smokers. Family conflict increased depression likelihood in never-smokers, in experimental smokers, and in current smokers.
Lorenzo-Blanco ^91^ 2016	United States	Longitudinal	1919	American Latino adolescents were surveyed based on their behavioral experiences (perceived discrimination, bullying victimization, social support, perceived school safety). Profiles characterized by high perceived discrimination and/or high bullying victimization in the absence of positive experiences had higher levels of depressive symptoms and higher risk of smoking.
Crane ^97^ 2015	United States	Longitudinal survey	1263	Baseline cigarette use was documented at age 15-16 years. Frequency of cigarette smoking and marijuana use, and depression symptoms were assessed at baseline, and then at 6, 15, 24, 60, and 72 months. Cigarette use frequency and depression symptoms were associated with frequency of marijuana use among males, but not in females. Frequency of marijuana use was associated with increased cigarette use frequency, especially among males who had higher symptoms of depression, but not in females.
Orlando ^99^ 2001	United States	Longitudinal	2,961	Data were collected from 2,961 adolescents. For both boys and girls, emotional distress in grade 10 was associated with increased smoking in grade 12. Similarly, smoking in grade 12 was associated with increased emotional distress in young adulthood.
Crone ^110^ 2007	United States	Longitudinal survey	1789	Psychosocial problems (behavioral and emotional) were measured at age 13 and again 2 years later to assess the association with smoking behavior in adolescence. Externalizing problems at baseline predicted the onset of smoking 2 years later. Internalizing problems only predicted smoking among girls. Reversibly, smoking at baseline was only associated with the onset of externalizing problems 2 years later.
Riehm ^112^ 2019	United States	Longitudinal survey	7702	Internalizing and externalizing problems were assessed for associations with initiation of e-cigarette, combustible cigarette, and dual-product use among adolescents. Adolescents with high externalizing problems were more likely to initiate use of e-cigarettes, combustible cigarettes, and both products. Adolescents with high internalizing problems were at increased risk of initiating use of e-cigarettes but not combustible cigarettes or both products.

PRD = perceived racial discrimination.

## Electronic cigarettes and depression

There has been an exponential increase in the utilization of e-cigarettes (electronic nicotine delivery systems) among adolescents, escalating from around 7.5% in 2007 to 20.8% in 2018.^113 , 114^ In 2018, these active users increased to 3.62 million, making e-cigarettes the most common tobacco source amongst adolescents.^114^ Moreover, e-cigarette use is also implicated in an estimated six times increased risk of initiation of conventional cigarette use among adolescent populations.^115^

E-cigarettes were introduced onto the market in 2007 and since then researchers have investigated their use and health risks. Leventhal and Zvolensky^116^ reported insufficient development of coping mechanisms and decreased problem solving and strategizing skills in adolescents with prolonged e-cigarette use. Progressive increase in depressive symptoms is also associated with unremitting e-cigarette consumption for more than a year.^117^ In their study based on the Youth Risk Behavior Survey (YRBS), Chadi et al.^118^ reported increased odds of depressive symptoms (adjusted OR: 1.37, 95%CI 1.19-1.57) among adolescents who were current e-cigarette users. Similarly, Lechner et al.^101^ explored the bidirectionality of e-cigarette use and depression among adolescents, observing that depressive symptoms were associated with e-cigarette initiation (OR: 1.015, 95%CI 1.003-1.023), while persistent use of e-cigarettes predicted increase in depressive symptoms over time. Many e-cigarette consumers are also found to have an increased prevalence of internalizing disorders including depression, anxiety, panic and obsessive-compulsive disorder compared to non-users, but less than was observed in dual and conventional cigarette users.^119^ Riehm et al.^112^ used Population Assessment of Tobacco and Health Study survey data to observe that adolescents with high externalizing and internalizing symptoms were more likely to initiate e-cigarette usage (adjusted relative risk ratio [aRRR]: 2.78; 95%CI 1.760-4.40 and aRRR: 1.61; 95%CI 1.12-2.33, respectively). However, the longitudinal study by Bandiera et al.^98^ examining the relationship between e-cigarettes use and depression observed that depressive symptoms predicted use of e-cigarettes at 6 months and 1 year, whereas use of e-cigarettes was not associated with development of depressive symptoms over time. Research on e-cigarette use and depression is mostly cross-sectional and/or of short duration, therefore, further long-term research is required to preclude any causal inferences and to investigate the individual characteristics associated with e-cigarette use and depression among the adolescent population.^69 , 70 , 120 - 122^

## Current evidence and future directions

There is much evidence in the current literature elucidating the intricate relationship between smoking and depression in the adolescent population. However, despite various studies reporting significant associations, the evidence on the causal relationship is still ambiguous. The heterogeneous observations in the literature lack concrete evidence on the directionality and the cause-effect relationships of smoking preceding depression or depression preceding smoking. Additionally, the relationship is made more complex by various external factors confounding the chronological order. Moreover, with the continuous rise in mental health conditions and smoking behavior, especially with recent rapid indulgence in newer smoking products among young adolescents, it is imperative to understand the causal link between the two conditions and untangle the influencing determinants.

Although many studies have explored this relationship using both cross-sectional and longitudinal study designs, substantial evidence on preceding associations has been reported by studies employing longitudinal data. Cross-sectional studies provide a snapshot at one point, whereas longitudinal study designs provide information over time. Moreover, sample selection in cross-sectional studies is limited by the presence of existing conditions. Therefore, longitudinal study designs involving individuals without diagnoses of depression and/or smoking behavior should be considered when investigating a causal association between the two conditions. Moreover, the literature also provides information on various biological and social confounders like genetics and family and peer influence, which significantly obscure this causal pathway and it is therefore important to recognize and control these factors when examining the association. It is also important to acknowledge that multiple studies used surrogate measures like sadness and affect change to evaluate depression instead of using validated scales based on DSM criteria. Therefore, the results of these studies should be interpreted with caution even when they report a significant association, as they may not reflect a true condition.

While it is essential to understand the limitations, it is also important to realize that there is a need for much research in this area. Of importance is to define a study population, which represents a homogenous sample of adolescents in the community to examine and control for the influencing mediators in the cause-effect pathway. It is also imperative to use validated instruments to measure depression and standardize metrics to quantify smoking behavior. Longitudinal data collection and serial sampling will represent the optimal strategy to ascertain the level of evidence for this causal relationship.

Finally, given the rises in both smoking behavior and depressive symptoms among adolescents, it is important to understand this association between smoking and depression. To do so will not only help educate young individuals to better understand the health consequences, but, more importantly, is also critical for designing effective treatment interventions, targeted clinical and behavioral therapies, and preventative public health strategies and policies.

## Conclusion

Although, the relation between smoking and depression is well established, the literature does not reflect the cause-effect relationship. Additionally, researchers have not yet been able to demonstrate concrete evidence on the direction of the association. This is a reflection of futile study designs, exclusion of confounding factors, and/or use of indirect measure of depression and quantification of smoking. Therefore, the association between depression and smoking should be explored further with rigorous longitudinal studies controlling for influencing factors to identify the causal association between the two conditions.
